# A structural *UGDH* variant associated with standard Munchkin cats

**DOI:** 10.1186/s12863-020-00875-x

**Published:** 2020-06-30

**Authors:** Ann-Kathrin Struck, Marina Braun, Kim Aline Detering, Peter Dziallas, Jasmin Neßler, Michael Fehr, Julia Metzger, Ottmar Distl

**Affiliations:** 1 Institute of Animal Breeding and Genetics, University of Veterinary Medicine Hannover (Foundation), 30559 Hannover, Germany; 2 Clinic for Small Animals, University of Veterinary Medicine Hannover (Foundation), 30559 Hannover, Germany

**Keywords:** Munchkin, Cat, *Felis catus*, Chondrodysplasia, Whole genome sequencing, *UGDH*

## Abstract

**Background:**

Munchkin cats were founded on a naturally occurring mutation segregating into long-legged and short-legged types. Short-legged cats showed disproportionate dwarfism (chondrodysplasia) in which all four legs are short and are referred as standard Munchkin cats. Long-legged animals are referred as non-standard Munchkin cats. A previous study using genome-wide single nucleotide polymorphisms (SNPs) for genome-wide association analysis identified a significantly associated region at 168–184 Mb on feline chromosome (FCA) B1.

**Results:**

In this study, we validated the critical region on FCA B1 using a case-control study with 89 cats and 14 FCA B1-SNPs. A structural variant within *UGDH* (NC_018726.2:g.173294289_173297592delins108, *Felis catus 8.0*, equivalent to NC_018726.3:g.174882895_174886198delins108, *Felis catus 9.0*) on FCA B1 was perfectly associated with the phenotype of short-legged standard Munchkin cats.

**Conclusion:**

This *UGDH* structural variant very likely causes the chondrodysplastic (standard) phenotype in Munchkin cats. The lack of homozygous mutant phenotypes and reduced litter sizes in standard Munchkin cats suggest an autosomal recessive lethal trait in the homozygote state. We propose an autosomal dominant mode of inheritance for the chondrodysplastic condition in Munchkin cats.

## Background

Short-legged cats have been reported as early as 1944, describing individuals with unusually short forelimbs but long-legged hind limbs, resulting in forelimb micromelia [1–3]. Today’s Munchkin cats presumably have their roots in a female cat with all four short legs on which Sandra Hockenedel [4] used to found the breed in 1983. This short-legged animal gave birth to a healthy litter, with half of the kittens showing short legs. This short-legged female cat and one of its short-legged male kittens established today’s Munchkin cat breed. To expand the Munchkin cats´ gene pool, these two Munchkin cats were mated to long-legged cats. Through this outcross program, a variety of coat colors and patterns as well as different Munchkin cat subtypes were developed [4–10] (Additional file 1). Breeders refer to short-legged as standard Munchkin and long-legged as non-standard Munchkin cats [4]. Offspring from standard Munchkin cat parents can segregate into standard and non-standard Munchkin cats. Munchkin cats have been recognized by The International Cat Association (TICA) as an independent breed since 1994. Breeding data collected by TICA suggest a dominant mode of inheritance [11, 12] and lethality in the homozygous state at an early embryonic stage [5].

The Munchkin cat phenotype and genotype is not yet fully characterized. A genome-wide association study (GWAS) was performed for the standard Munchkin phenotype resulting in a highly significant critical region at 168–184 Mb on feline chromosome (FCA) B1 [13].

The present study characterized the standard Munchkin phenotype using computed tomography (CT) scans and comparative long bone measurements. Pedigree data of Munchkin cats were collated to establish the mode of inheritance. In order to unravel the responsible mutation for standard Munchkin cats, we analyzed whole genome sequencing (WGS) data of a Munchkin cat family and controls for Munchkin-associated variants. Candidate variants were validated in large cohorts of standard and non-standard Munchkin cats as well as in a cat breed panel with the most popular breeds. We identified a structural *UDP-glucose 6-dehydrogenase* (*UGDH*) variant perfectly segregating with the standard Munchkin cat phenotype. For this variant, we assumed dominance as homozygous mutants were not observed.

## Results

### Munchkin cat phenotype

**Fig. 1:**
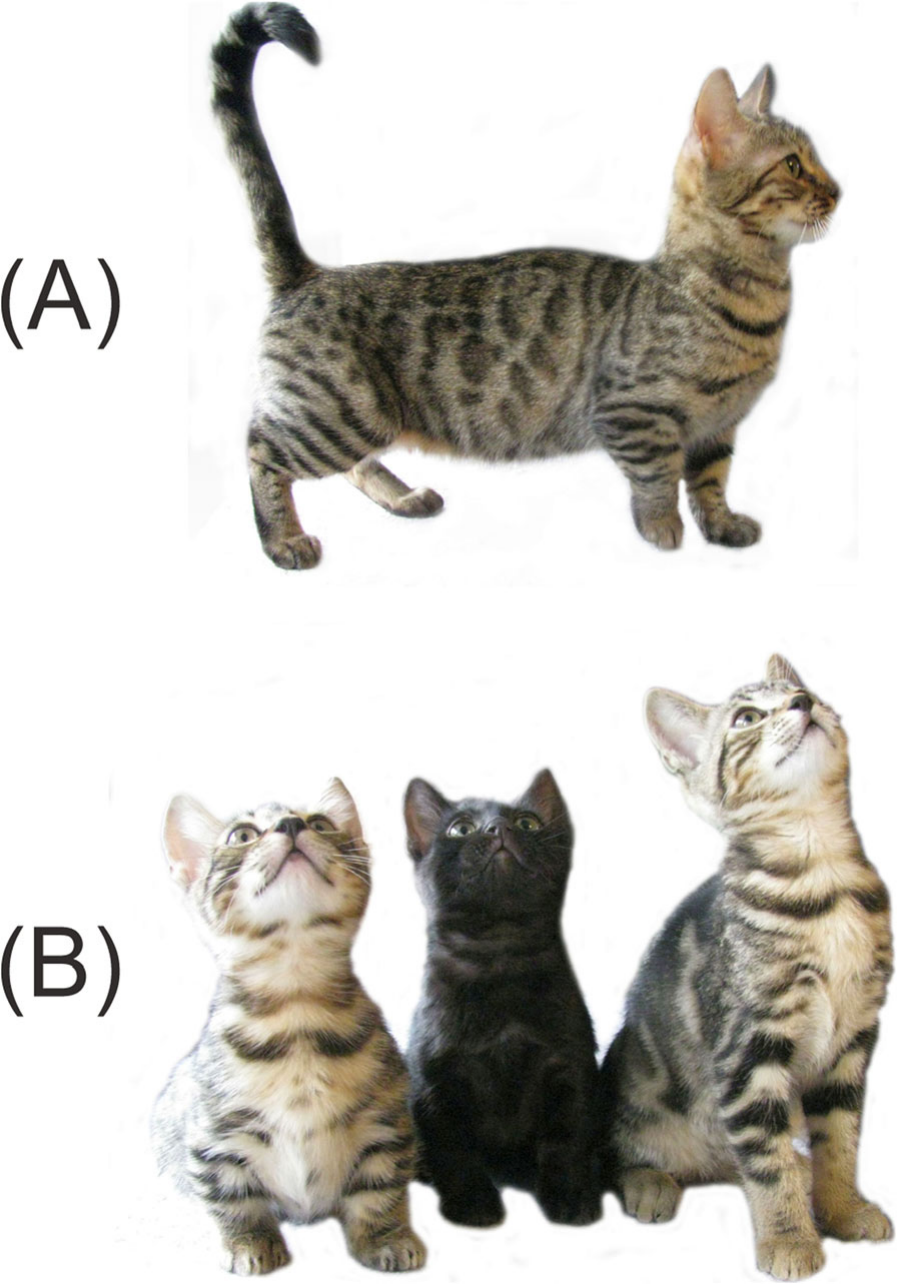
Munchkin cat phenotype. **a** Lateral view of the 4-year old standard Munchkin cat sire. Note the head is proportional to the body but the legs are shortened. **b** Frontal view of the kittens from litter A showing two male standard Munchkin kittens (right) and one female non-standard Munchkin full sibling (left)

The standard Munchkin cats of the subtype Genetta showed a disproportionate short stature with shortened fore- and hind limbs compared to a female non-standard Munchkin cat (Fig. 1). Subtype Genetta resulted from the outcrossing program of standard Munchkin with Bengal cats and this subtype is recognized by TICA as an experimental breed. The upper- and forearms of the front limbs as well as the upper- and forelegs had nearly the same length in the standard Munchkin cats. The size of paws was proportional to the body. The head of both types, standard and non-standard Munchkin cats showed no signs of chondrodysplastic changes such as brachygnathia superior or a spherical appearance.

**Fig. 2:**
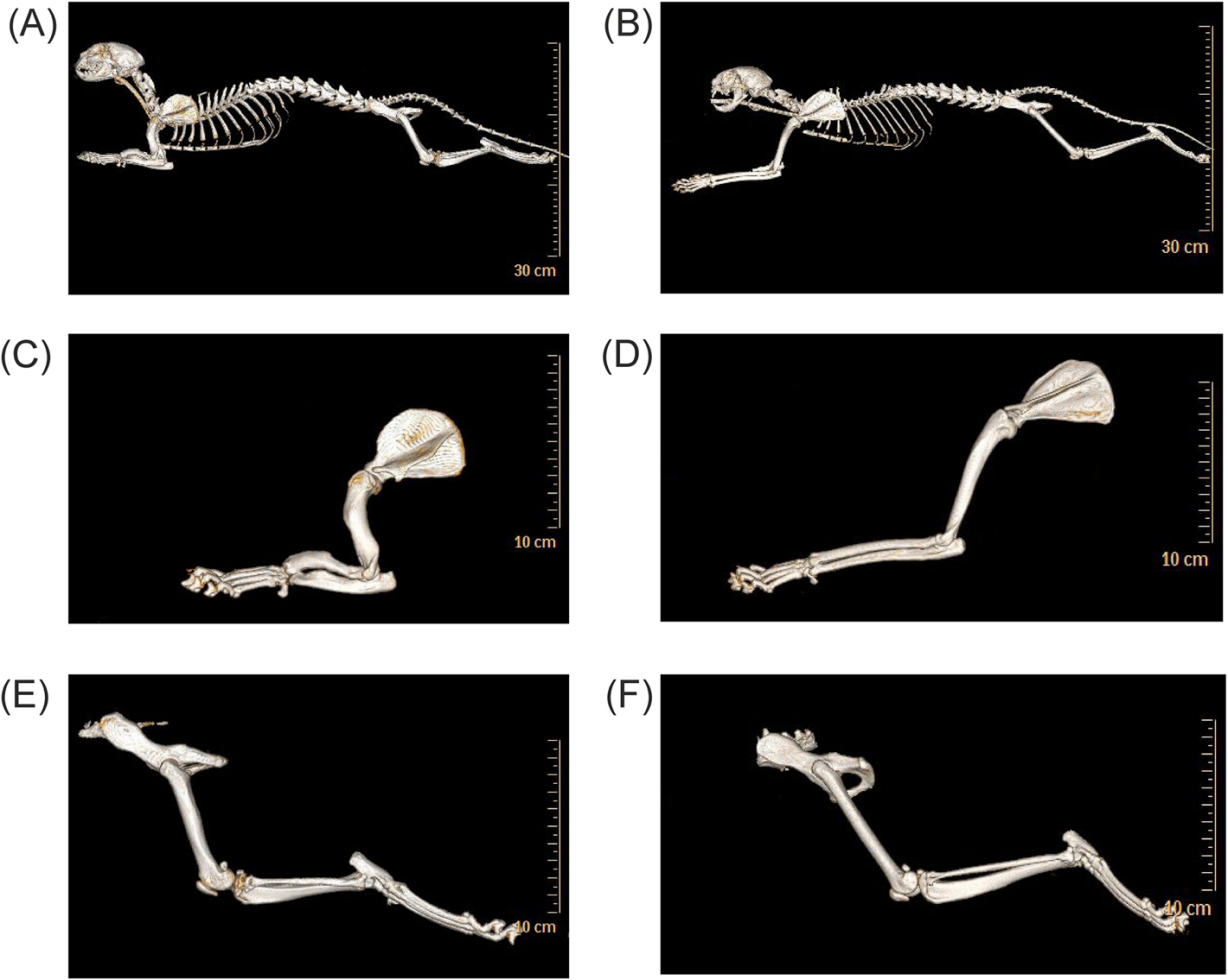
Computed tomography of a 4-year old standard Munchkin cat sire and an adult female domestic cat control. Lateral view of a standard Munchkin cat (**a**, **c**, **e**) and a domestic cat (**b**, **d**, **f**). The limbs of the standard Munchkin cat are shortened in relation to the body. The fore limbs of the standard Munchkin cat (**c**) show a shortening of all distal and proximal long bones and higher diaphyseal diameters, particularly of *humerus*, compared to the fore limbs of domestic cat (**d**). The hind limbs (**e**, **f**) also show higher diaphyseal diameters in particular in the femur as well as a shortening of the long bones

CT of the forelimbs of the 4-year old standard Munchkin tomcat showed a shortening of ​​all distal und proximal long bones including *humerus*, *radius*, *ulna* and *metacarpalia* (Fig. 2 and Additional file 2). Lengths of the *humerus, radius*, *ulna, metacarpalia, femur* and *tibia* of the standard Munchkin cat were reduced by 71, 58, 64, 84, 74, and 70%, respectively, in comparison to the domestic cat. *Metatarsalia* had normal length. All bones of the front and hind limbs exhibited higher average diaphyseal diameters, in particular *humerus* (+ 14%) and *femur* (+ 29%). Furthermore, the *humerus* showed a slight internal rotation along its longitudinal axis, resulting in a moderate incongruity in the elbow joint with axial deviation. The *humerus compacta* in the middle segment revealed a moderate degree of thickening of 2.4 mm (0.09 in). Furthermore, the *radius* was too short in relation to the *ulna*, as well as the *ulna* was slightly medially rotated, whereas the *radius* was bent to a high degree in the longitudinal axis of about 43°. This resulted in an incongruity in the ulnocarpal and radiocarpal joint. The hind limbs did not show any rotation and structural effects.

### Pedigree analysis

Pedigree analysis of the Munchkin cat family of the subtype Genetta demonstrated that three repeated matings between a standard Munchkin tomcat and a standard Munchkin queen resulted in seven standard Munchkin and two non-standard Munchkin cats (Additional file 3). As reported by breeders, matings among standard Munchkin cats resulted in reduced litter sizes.

### Association and haplotype analysis

Association analysis for 14 SNPs on FCA B1 gave significant *P*-values for 9/14 SNPs (Additional file 4). These nine significant SNPs were located in the critical region FCA B1. The SNPs B1_9 (g.173759872A > G) and B1_10 (g.174407393 T > C) reached the highest P-values for both the genotype (B1_9, *P* = 9.17^− 18^; B1_10, *P* = 5.79^− 18^) and allele (B1_9, *P* = 7.59^− 17^; B1_10, *P* = 6.33^− 17^) distribution among cases and controls. Haplotype analysis corroborated the significant association for the single SNPs (Additional file 5).

### Whole genome sequencing and variant detection

A mean read coverage of 11.99X (non-standard Munchkin kitten), 12.67X (standard Munchkin kitten), 12.90X (standard Munchkin sire) and 8.49X (standard Munchkin dam) was obtained in WGS data. A filter analysis for exclusively heterozygous genotypes in the standard Munchkin cats and homozygous wild genotypes in 16 control cats did not reveal functional variants on FCA B1, B4 and X.

Thus, an obvious protein modifying candidate variant was not identified in the genome-wide associated region on FCA B1 at 168 to 184 Mb or within the genomic regions with the next highest peaks on FCA B4 and X from the previous GWAS [13].

Structural variant analysis with LUMPY software revealed one variant on FCA B1 harbouring a heterozygous deletion of 3303 bp in all three standard Munchkin cats but homozygous wild type in the non-standard Munchkin kitten and all 16 controls. The 3303 bp deletion was located within *UDP-glucose 6-dehydrogenase* (*UGDH*) at 173,294,289-173,297,592 bp (*Felis catus 8.0*; NC_018726.2:g.173294289_173297592delins108), equivalent to 174,882,895-174,886,198 bp (*Felis catus 9.0*; NC_018726.3:g.174882895_174886198delins108) on FCA B1 within the critical region. This 3303 bp deletion comprises 1191 bp of intronic sequences (intron 10–11, ENSFCAT00000009602.6 or intron 9–10, ENSFCAT00000055794.2), exon 11 (ENSFCAT00000009602.6) or 10 (ENSFCAT00000055794.2) and 2001 bp of the 3′-UTR (Additional file 6).

### Sanger sequencing

Sanger cDNA sequencing confirmed the break points of the deletion predicted in the LUMPY software. Furthermore, an insert of 108 bp was identified between the break points of the deletion in all three standard Munchkin cats (5′-AAATCACTAAATCACTAAATAAAAGTTCAAAGATATGAACATAGATATGAATGACTACCAGAGAAGGAGATTTGCTTCCCGGATTATAGAGAGATTATTTAGAGATTA-‘3). An NCBI Blast search (https://blast.ncbi.nlm.nih.gov/Blast.cgi?PAGE_TYPE=Blast Search) of this insert against feline reference genomes (*Felis catus* 8.0 and *Felis catus* 9.0) did not identify any regions with significant sequence homology. However, two sequence similarities (100%) of 51 bp (max score 95.3, E-value 8 × 10^–18)^) were identified on FCA B1. The first one, within *UGDH* distal to the indel in the exonic region at 173,290,767-173,290,817 bp (*Felis catus 8.0*) and 174,879,349- 174,879,399 bp (*Felis catus 9.0*) and the second, in the intergenic region of ENSFCAG00000031593 at 134,584,870- 134,584,920 bp (*Felis catus 9.0*).

### Mutation analysis

We did not detect variants in wild type cats for the cDNA sequence of exon 10 and 11 (ENSFCAT00000009602.6) or exon 9 and 10 (ENSFCAT00000055794.2) and the proximal parts of the 3’UTR using primer pairs within exon 10 and 3’UTR (PCR-type 3 and PCR-type 1, Additional file 7). Amplicons of cDNA from wild type cats were of an expected size of 2514 bp (PCR-type 1) or 185 bp (PCR-type 3). Sanger sequencing of wild type allele cDNA for a region including exon 10 (ENSFCAT00000009602.6) or exon 9 (ENSFCAT00000055794.2) and 3’UTR (PCR-type 4–7) confirmed the 3’UTR region reference genome sequence (Ensembl database).

**Fig. 3:**
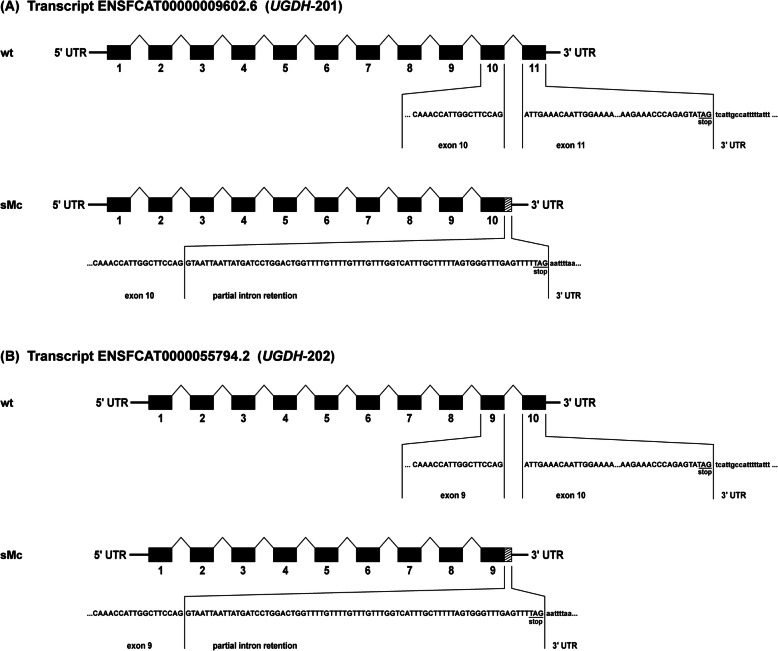
Schematic representation of cDNA in wild type (wt) and standard Munchkin cats (sMc) for PCR-type 1. PCR across the deletion from (**a**) exon 10 (ENSFCAT00000009602.6) or (**b**) exon 9 (ENSFCAT00000055794.2) to the 3′-UTR (non-deleted region) showed a 493 bp amplicon. Sanger sequencing of this 493 bp amplicon showed that the 108 bp insert is not transcribed but this PCR-product consists of 73 bp of the exon 10 (ENSFCAT00000009602.6) or exon 9 (ENSFCAT00000055794.2), plus a partially retained intron 10–11 (ENSFCAT00000009602.6) or intron 9–10 (ENSFCAT00000055794.2) of 309 bp and 115 bp of the 3’UTR

PCR-type 2 was designed to show whether the 108 bp insert (see above) is transcribed into mRNA and in this case, should give an amplicon size of 110 bp only for the mutant allele in standard Munchkin cats. In standard Munchkin cats heterozygous for the NC_018726.3:g.174882895_174886198delins108 (*Felis catus 9.0*) mutation, PCR-type 1 should yield two amplicons with sizes of 402 bp (insertion not transcribed) or 510 bp (insertion transcribed) and 2514 bp for the mutant and wild type allele, respectively. The shorter fragment size of 402 bp was calculated based on genomic DNA analysis assuming deletion of exon 11 (ENSFCAT00000009602.6) or exon 10 (ENSFCAT00000055794.2) and 2001 bp of the proximal part of the 3’UTR. However, PCR-type 1 yielded an amplicon size of 493 bp for the mutant allele in standard Munchkin cats. This amplicon contained 73 bp of exon 10 (ENSFCAT00000009602.6) or exon 9 (ENSFCAT00000055794.2) and 309 bp from intron 10–11 (ENSFCAT00000009602.6) or intron 9–10 (ENSFCAT00000055794.2) and in addition, 111 bp of the 3’UTR (Fig. 3 and Additional file 8). We found an alternative splice site in the intronic sequence with the motif TACACAatttaaaa explaining the partial intron retention of 309 bp. PCR-type 2 failed to generate an amplicon and thus, suggests that the 108 bp insert is not transcribed into mRNA.

Open reading frame finder (ORF, http://www.ncbi.nlm.nih.gov/projects/gorf/) prediction for the mutated cDNA sequence predicted an UGDH protein truncated by 12 aa (Additional file 9). For non-standard and wild type cats, an UGDH wild type protein of 494 aa (*UGDH*-201, ENSFCAT00000009602.6) and 427 aa (*UGDH*-202, ENSFCAT00000055794.2) was predicted. The partial intron retention was predicted to result in an elongation of exon 9 (ENSFCAT00000055794.2) or exon 10 (ENSFCAT00000009602.6) by 75 bp with a new stop codon (Additional file 8), as well as a modified 3’UTR sequence starting within the partially retained intronic sequence. The proximal 218 bp of the wild type 3’UTR were found to be missing in the mutated allele according to our cDNA Sanger sequences. Based on *Felis catus 9.0*, the predicted protein sequence modification (start at aa 459 for ENSFCAT00000009602.6 or aa 392 for ENSFCAT00000055794.2) was found to be located within the UDP-glucose 6-dehydrogenase (PTHR11374:SF35, PANTHER), UDP-glucose dehydrogenase/UDP-mannac dehydrogenase (PTHR11374, PANTHER), UDPglc_DH_euk (PIRSF500133, PIRSF) and UDPglc_GDPman_dh domain (PIRSF000124, PIRSF) (Additional file 10). Comparative species alignments with Clustal Omega (*UGDH*-201, ENSFCAT00000009602.6) located the indel in a highly conserved region of *UGDH* (Additional file 11).

### Validation

The *UGDH* structural variant comprising a deletion and an insertion on FCA B1 was found in all 42 standard Munchkin cats whereas the 15 non-standard Munchkin cats and all 203 cats of other breeds were homozygous wild type. Perfect segregation of the *UGDH* structural variant with the standard and non-standard Munchkin cats was shown for the Munchkin cat family of the subtype Genetta (Additional file 3).

## Discussion

This is the first study identifying a mutation in *UGDH* on FCA B1 perfectly associated with standard Munchkin cats exhibiting a disproportionate stature with shortened legs. In our study, we confirmed the significantly associated genome region on FCA B1 at 168–184 Mb [13] in a case-control and haplotype analysis and identified a potential causative structural variant in *UGDH.* UGDH is involved in heparan sulfate proteoglycan (HSPGs) synthesis through catalyzing conversion of UDP-glucose into UDP-glucuronic acid (UDP-GlcA) [14]. Proteoglycans regulate growth factors and morphogens such as fibroblast growth factor (Fgf), hedgehog (Hh), transforming growth factor-ß (TGFß) and wingless (Wg, Wnt family) [14–17]. Studies on growth related signaling molecules showed Wnt and Fgf proteins necessary for limb structure, ensuring proliferation, differentiation and cell survival [18, 19]. Various mutations in Wnt proteins (Wnt-3a and Wnt-7a) were found to result in abnormalities during limb formation including irregular limb size [17, 20]. Furthermore, a disruption of *fgf10* in mutant zebrafish (daedalus) was shown to provoke an absence of *fgf10* activity and thus, a severely compromised fin bud development [19]. Whether the standard Munchkin cat fits into a model with disturbed proteoglycan signaling and aberrant signals for morphogens requires further studies. Furthermore, the finding that no homozygous mutant individual was present in the study population supported the previous suggestion of lethality of homozygous mutant individuals at an early embryonic stage [5].

Our investigations of heterozygous mutant standard Munchkin cats compared to homozygous wild type cats showed a partial retention of the last intron in *UGDH* resulting in a loss of an acceptor site and a new alternative donor site. cDNA sequencing results revealed a partial intron retention of 309 bp as well as a predicted UGDH protein with a truncation of 12 aa and a modification of the aa sequence. Previous studies showed that intron-retaining transcripts are prone to produce novel protein isoforms with modified biological functions resulting in detrimental effects on cells and tissues [21–23]. This was also observed in Miniature Zebu, in which an *aggrecan (ACAN)*-associated mutation caused a recessive lethal chondrodysplasia due to a truncated and modified protein [24]. In Dexter cattle, a functionally null variant of *ACAN* was found in a homozygous state in spontaneously aborted fetuses and non-viable calves with a bulldog-like phenotype [25]. In our study, we propose the predicted truncation and modification of UGDH as the reason for an early embryonic death in a homozygous mutant state. Studies for *UGDH* in mice and drosophila (*sugarless or kiwi*) mutant embryos deficient for UGDH activity showed also embryonic lethality [26–30]. The zebrafish *ugdh* mutant jekyll was found to be lethal due to severe defects during organogenesis of the heart [28]. Heterozygous standard Munchkin exhibit reduced leg length without impaired health, as found in Dexter cattle [25].

## Conclusions

In conclusion, we identified a structural *UGDH* variant perfectly associated with the standard Munchkin phenotype. No homozygous mutant cats have been observed, supporting the suggestion that the mutation has an autosomal dominant mode of inheritance for the chondrodysplastic condition in Munchkin cats. A diagnostic test is now available for cat breeders to prove if their animals carry the standard Munchkin cat mutation in order to get recognition by TICA.

## Methods

### Animals

Genomic DNA was isolated from EDTA-blood, hair root or tissue samples. We sampled 42 standard Munchkin and 15 non-standard Munchkin cats from several subtypes (Additional file 12). Standard Munchkin cats included the subtypes Genetta (*n* = 10), Napoleon (*n* = 2), Dwelf, (*n* = 1), Bambino (*n* = 10), Lambkin (*n* = 1), longhaired (*n* = 11) and shorthaired (*n* = 7). Non-standard Munchkin cat samples were available for the subtypes Genetta (*n* = 2), Bambino (*n* = 6), longhaired (*n* = 1) and shorthaired (*n* = 6). Cats of the subtype Genetta belonged to a family segregating into long-legged and short-legged types (Additional file 3). The sire and three of his kittens (two standard and one non-standard Munchkin cats) underwent a detailed clinical examination. The parents and two full sibling kittens from this segregating litter were chosen for WGS. For controls, we sampled 203 non-Munchkin cats representing British shorthair, Bengal, Ragdoll, Exotic Shorthair, Don Sphinx, Birman, Chartreux, Maine Coon, Norwegian Forest, Persian, Russian Blue, Scottish Fold, Siamese, Siberian, Turkish Angora, Oriental Shorthair, and domestic shorthair.

### Clinical examination

For the clinical investigation, members of the segregating Munchkin cat family of the subtype Genetta were available. A cat breeder provided a 4-year old standard Munchkin tomcat and three of his progeny including two male standard and a female non-standard Munchkin kitten for a detailed examination. The 4-year old standard Munchkin tomcat underwent a CT scan in sternal recumbency using a multislice helical CT scanner (Brilliance 64-CT, Philips Medical Systems, Best, The Netherlands). A slice thickness for the head of 0.67 mm (0.026 in) and settings of 120 kV/ 250 mAs and for the body a slice thickness of 0.67 mm (0.026 in) and settings of 140 kV/ 250 mAs were set for analysis. A 1024 × 1024 matrix was used.

In addition, absolute total length, diaphyseal length and the diaphyseal diameter of *humerus*, *radius*, *ulna*, *femur*, *tibia, metacarpalia* and *metatarsalia* bones were measured according to Jones and Jolly [31]. The relative length of individual bones was determined through the ratio of length of the respective individual bone to the length measured from the proximal end of the *humerus* to the distal end of *metarcarpus III*.

### Haplotype analysis

To refine the critical genome-wide associated region on FCA B1, Kompetitive Allele Specific PCR (KASP) assay was used (LGC Genomics, Middlesex, UK). We selected 14 SNPs (PRJEB30080) of which four SNPs were located distally to the critical region and ten SNPs within the genome-wide associated region at a distance among each other of about 430 to 7600 kb on FCA B1. These 14 SNPs were genotyped in 9 standard Munchkin cats, one non-standard Munchkin cat and 79 controls. These animals were unrelated. KASP genotyping was performed according to standard protocols (LGC) using an ABI7300 real-time system for 96 well plates (Additional file 13). A case-control analysis was carried out using SAS/Genetics, version 9.4 (Statistical Analysis System, Cary, NC, USA). Haplotype analysis was done with the procedure HAPLOTYPE in SAS.

### Whole genome sequencing

Animals for WGS were from the Munchkin cat family of the subtype Genetta including the standard Munchkin parents (sire and dam) and two members of their litter with a standard Munchkin cat male and a non-standard Munchkin cat female (Additional file 3). Libraries were prepared using the NEBNext Ultra II DNA Library Prep Kit for Illumina (New England BioLabs, Ipswich, MA, USA) and run on an Illumina NextSeq500 in a 2 × 150 bp paired-end mode. Quality control was performed using fastqc 0.11.5 [32] and reads were trimmed using PRINSEQ (V 0.20.4) [33].

WGS data was mapped to the cat reference genome *Felis catus 8.0* (Ensembl) using BWA 0.7.13 [34]. Sorting, duplicate marking and indexing was performed using Picard tools (http://broadinstitute.github.io/picard/, version 2.9.4) and SAMtools 1.3.1 [35]. For final variant calling GATK version 3.7 [36] with the Base Quality Score Recalibrator (BQSR), Haplotype Caller [37] and Variant Recalibrator were used. Variant calling data were compared to WGS data from 16 different individuals including Bengal (SAMN05980341, SAMN05980358), Siamese (SAMN05980342), Peterbald (SAMN05980359, SAMN14103114), Oriental shorthair (SAMN14103123, SAMN14103124, SAMN14103127), and domestic shorthair (SAMN14103116, SAMN14103119, SAMN14103120, SAMN14103121, SAMN05980374, SAMN05980340, SAMN05980323, SAMN05980325).

A read depth of 8–999 and quality score values > 20 were applied for variant detection. Those variants, which were heterozygous in the three standard Munchkin cats, homozygous wild type in the non-standard Munchkin cat and homozygous wild type in all controls and concordant to an autosomal monogenic dominant inheritance, were filtered using SAS (version 9.4, Statistical Analysis System, Cary, NC, USA) for further investigation. Only variants with high or moderate effects according to SNPEff version 4.3 t (2017-11-24, SNPEff database *Felis catus8.0*) [38] were considered for further analysis. A candidate gene list from previous studies about chondrodysplasia was used to find candidate genes in the filtered variant list [24].

### Structural variant detection

For structural variant detection, LUMPY software, version 0.2.13 [39], integrating multiple structural variation signals jointly across multiple samples, was run for Bam files of all four Munchkin cats and 16 controls. The generated VCF file was filtered for structural variants on FCA B1, heterozygous in the standard Munchkin cats and homozygous wild type in the non-standard Munchkin cat and the 16 controls.

### Mutation analysis

To test whether the 108 bp insert is transcribed or not transcribed into mRNA, we isolated RNA from hair roots stabilized in RNAlater reagent (Qiagen, Hilden, Germany) of two standard Munchkin cats of the subtype Genetta and two domestic shorthair cats. All samples were transcribed into cDNA according to standard protocols (Maxima First Strand cDNA Synthesis Kit, Thermo Fisher). Primer pairs were designed using Primer3 tool (version 0.4.0, http://bioinfo.ut.ee/primer3-0.4.0/). One primer pair was designed to produce a PCR-product in mutant and wild type allele (PCR-type 1), one primer pair to produce a PCR-product only if the mutant allele is present and the insertion is transcribed (PCR-type 2), and one control primer pair to produce a PCR-product in the presence of wild type allele (PCR-type 3, Additional file 14). The first primer pair (PCR-type 1), located across the deletion from exon 10 (ENSFCAT00000009602.6) or exon 9 (ENSFCAT00000055794.2) to the 3′-UTR within the non-deleted region, yields an expected amplicon size of 510 bp for the mutant allele if the insertion is transcribed and 402 bp for the mutant allele if the insertion is not transcribed, as well as 2514 bp for the wild type allele (Additional file 7). The second primer pair (PCR-type 2) from the proximal region of exon 10 (ENSFCAT00000009602.6) or exon 9 (ENSFCAT00000055794.2), to a region within the 108 bp insert produces an expected amplicon of 110 bp only in the mutant allele when the insertion is transcribed. This PCR-product is not present in the wild type or mutant allele when the insertion is not transcribed. The third primer pair (PCR-type 3) was designed to bridge exon 10 and 11 (ENSFCAT00000009602.6) or exon 9 and 10 (ENSFCAT00000055794.2) with an expected amplicon size of 185 bp for the wild type allele in standard Munchkin cats and control cats with wild type alleles on both chromosomes. Furthermore, we designed five primer pairs (PCR-type 4–7) for cDNA sequencing the region spanning exon 10 (ENSFCAT00000009602.6) or exon 9 (ENSFCAT00000055794.2) and those parts of the 3’UTR present in the mutant and wild type allele (Additional file 15). PCR included 0.25 μM of the respective primers, 20 ng cDNA, 12.5 μl UCP HiFidelity MasterMix (Qiagen, Hilden, Germany) and was prepared as recommended by Qiagen, Hilden, Germany. Sanger sequencing was done for the standard Munchkin dam from the Munchkin cat family of the subtype Genetta (Additional file 3) for PCR-type 1 and in one domestic shorthair cat for PCR-type 4–7.

In addition, open reading frames were predicted using ORF Finder (http://www.ncbi.nlm.nih.gov/projects/gorf/). Protein domains predicted by InterProScan (http://www.ebi.ac.uk/interpro/search/sequence-search) for PANTHER [40] and PIRSF [41] were obtained by Ensembl database (protein summary) [42]. For comparative species alignments Clustal Omega (https://www.ebi.ac.uk/Tools/msa/clustalo/) was used [43]. Primer blast was done using NCBI nucleotide blast (https://blast.ncbi.nlm.nih.gov/Blast.cgi? PROGRAM = blastn&PAGE_TYPE = BlastSearch&LINK_LOC = blasthome). The sequence of the 108 bp insertion was compared to the reference genomic sequences *Felis catus 8.0* and *Felis catus 9.0*. The sequence similarity, highest alignment score and the expected value were calculated.

### Validation

A structural variant composed of a 3303 bp deletion (ss5015497294) and a 108 bp insertion (ss5015497295) within the critical region on FCA B1 was Sanger sequenced and visualized using a duplex PCR in individuals including standard Munchkin, non-standard Munchkin and control cats. Duplex PCR was performed in 260 individuals including 15 non-standard Munchkin, 42 standard Munchkin and 203 controls from breeds other than Munchkin cats. Sanger sequencing was done in the standard Munchkin sire, dam and the standard Munchkin kitten. A forward primer proximally of the detected structural variant and two different reverse primers located within the deleted region and distal of this deletion were designed using Primer3 tool (version 0.4.0, http://bioinfo.ut.ee/primer3-0.4.0/) (Additional file 16 and Additional file 17). Duplex PCRs were performed in 22-μl reaction volumes containing 2 μl DNA, 1.5 mM deoxyribonucleoside triphosphates, 5 pmol of primers MK_del_R and MK_wt_R, 10 pmol of primer MK_wt_F, 1.5 U of *Taq* polymerase in the reaction buffer supplied by the manufacturer (MP Biomedicals, Eschwege, Germany) and 4.2 μl enhancer reagent (MP Biomedicals). After a 5 min initial denaturation at 95 °C, 40 cycles of 30 s at 94 °C, 30 s at 59 °C, and 45 s at 72 °C were run on a Thermocycler TProfessional 96 (Biometra, Göttingen, Germany). All samples were visually evaluated on a 1% agarose gel using Gel iX20 Imager (Intas Science Imaging Instruments, Göttingen, Germany).

Break point validation was performed in the three standard Munchkin cats of the subtype Genetta with WGS data using the primers MK_wt_F and MK_del_R accoding to the duplex PCR protocol. Evaluation of Sanger sequences was performed using Sequencher 4.8 software (GeneCodes, Ann Arbor, MI, USA).

## Supplementary information

**Additional file 1.** Survey on subtypes of Munchkin cats created by outcrossing with different cat breeds.

**Additional file 2.** Comparison of the length measurements for the long bones using CT images. A 4-year old male standard Munchkin cat is compared with an adult female domestic cat. Total (TL) and relative leg lengths, diaphyseal lengths (DL), and diaphyseal diameters (DD) in centimeters (cm), and inches (in) of front and hind limbs are shown.

**Additional file 3 **Pedigree of Munchkin cat family of the subtype Genetta. Pedigree of the Munchkin cat family of the subtype Genetta segregating into long-legged and short-legged types. Furthermore, the pedigree shows that all standard Munchkin cats are heterozygous for the *UGDH-*indel (NC_018726.2:g.173294289_173297592delins108, *Felis catus 8.0*, equivalent to NC_018726.3:g.174882895_174886198delins108, *Felis catus 9.0*) and all typed non-standard Munchkin cats were homozygous wild type. For 32 cats, samples were not available and not typed.

**Additional file 4 **Case-control study for the standard Munchkin cat phenotype using 14 SNPs on feline chromosome B1. The SNPs B1_5 to B1_14 are located within the significantly genome-wide associated region at 168 to 184 Mb. SNP IDs with their nomenclature for *Felis catus* 8.0 and (*Felis catus* 9.0), odds ratios with their lower and upper confidence intervals (CL), χ^2^-square values for the genotypic and allelic distribution as well as the corresponding –log_10_*P*-values are shown. The most significant values are printed in bold.

**Additional file 5 **Haplotype analysis using SNPs B1_9 (g.173759872A > G) and B1_10 (g.174407393 T > C) as well as the indel (NC_018726.2:g.173294289_173297592delins108) on feline chromosome B1 (*Felis catus 8.0*). The haplotypes with their frequencies, χ^2^-statistics and P-values are shown.

**Additional file 6 **Gene models of *UGDH*. *UGDH* has two transcripts, (A) *UGDH*-201 harboring 11 exons and (B) *UGDH*-202 harboring ten exons according to Ensembl for *Felis catus 9.0*. The detected *UGDH-*indel (NC_018726.2:g.173294289_173297592delins108, *Felis catus 8.0*, equivalent to NC_018726.3:g.174882895_174886198delins108, *Felis catus 9.0*) is causing a loss of 1191 bp within the last intron, a complete loss of the last exon and a loss of 2001 bp of the 3′-UTR. Black boxes indicate exons. The chromosomal positions of the indel is given for *Felis catus 8.0* reference genome (*Felis catus 9.0* positions are in bold).

**Additional file 7 **Schematic representation of PCR-type 1–3 for cDNA in wild type (wt) and standard Munchkin cats (sMc) for (A) *UGDH*-201 and (B) *UGDH*-202 for *Felis catus 9.0*. The PCR-type 1 from exon 10 (ENSFCAT00000009602.6) or exon 9 (ENSFCAT00000055794.2) to the non-deleted 3′-UTR region should yield an expected amplicon size of 510 bp for the mutant allele in case the insertion is transcribed and a 402 bp for the mutant allele when the insertion is not transcribed as well as 2514 bp for the wild type allele. The PCR-type 2 from the proximal region of exon 10 (ENSFCAT00000009602.6) or exon 9 (ENSFCAT00000055794.2) to a region within the 108 bp insert produces an expected amplicon size of 110 bp for the mutant allele when the insertion is transcribed. PCR-type 3 from exon 10 to 11 (ENSFCAT00000009602.6) or exon 9 to 10 (ENSFCAT00000055794.2) produces an amplicon size of 185 bp in standard Munchkin cats and controls.

**Additional file 8 **DNA and cDNA sequences. The genomic DNA sequence of standard Munchkin cats (sMc) (A), the expected cDNA sequence based on this genomic DNA sequence (402 bp, B), as well as the validated Sanger sequence of cDNA with a confirmed amplicon size of 493 bp (PCR-type 1, C) is shown. The 493 bp amplicon comprises 73 bp of the exon 10/9 (green), followed by a partially retained intron 10–11/9–10 of 309 bp (blue) and 111 bp of the 3’UTR (black). The 108 bp insertion, which was not found in cDNA is given in pink. Start positions based on *Felis catus 8.0* (*Felis catus 9.0*) and the newly predicted stop codon (underlined) are displayed.

**Additional file 9 **Variant effect on protein sequence. The amino acid (aa) sequence of the two transcripts (*UGDH*-201, ENSFCAT00000009602.6 and *UGDH*-202, ENSFCAT000000557945.1) with the newly predicted stop codon are shown. Open reading frame prediction gives a 12-aa truncated UGDH protein. Wild type cat (wt) protein sequences are compared to standard Munchkin cat (sMc) sequences.

**Additional file 10 **Domains predicted by InterProScan. (A) Predicted protein domains of transcript *UGDH*-201 (ENSFCAT00000009602.6) and (B) *UGDH*-202 (ENSFCAT000000557945.1) based on *Felis catus 9.0* (Ensembl protein summary) are shown. Wild type cat (wt) protein length are compared to standard Munchkin cat (sMc) length.

**Additional file 11.** Comparative UGDH protein sequence alignment. Asterisks represent positions with a fully conserved residue, colons a conservation of strongly similar and periods weakly similar properties between different species. The indel is located within a highly conserved region of UGDH.

**Additional file 12.** Number of animals and breeds included in this study. Standard Munchkin cat subtypes, non-standard Munchkin cat subtypes and controls from various breeds are displayed.

**Additional file 13 **Primer pairs used for haplotype analysis. The single nucleotide variants located on chromosome B1 were genotyped using a Kompetitive Allele Specific PCR (KASP) assay. The SNP ID, nomenclature of *Felis catus 8.0* and *9.0*, accession numbers, sequences of forward and reverse primers, annealing temperature (AT), as well as the number of cycles are shown.

**Additional file 14 **Primer pairs used for complementary DNA amplification of *UGDH*. PCR was done to test if the 108 bp insert is transcribed or not transcribed based on *Felis catus 9.0*. PCR-type 1 produces an expected amplicon of 510 bp for the mutant allele if the insertion is transcribed, and a 402 bp for the mutant allele if the insertion is not transcribed, as well as 2514 bp for the wild type allele. PCR-type 2, spanning from exon 10 (UGDH-201 (ENSFCAT00000009602.6)) or exon 9 (UGDH-202 (ENSFCAT00000055794.2)) to the 108 bp insert, should have an expected amplicon size of 110 bp for the mutant allele if the insertion is transcribed. PCR-type 3 produces an amplicon with 185 bp in standard Munchkin cats, and controls for the wild type allele.

**Additional file 15.** Primer pairs used for complementary DNA amplification spanning exon 10 (ENSFCAT00000009602.6) or exon 9 (ENSFCAT00000055794.2) to 3’UTR. All PCR-products were present in the wild type allele. PCR-type, primer pairs, sequences of forward and reverse primer, annealing temperatures (AT), and expected product sizes (bp) are shown.

**Additional file 16.** Primer pairs used for validation and Sanger sequencing. Validation of the indel was done using duplex PCR (MK_wt_F, MK_wt_R and MK_del_R), amplicons for Sanger sequencing were generated with primer pairs MK_wt_F, and MK_del_R.

**Additional file 17 **Validation of deletion within *UGDH* region (*Felis catus 9.0*). A forward primer (MK_wt_F) proximally of the detected structural variant and two different reverse primers located within the deleted region (MK_wt_R) and distal of this deletion (MK_del_R) are shown. An amplicon of primers MK_wt_F and MK_del_R could only be observed in mutant allele, whereas the PCR failed for the wild type allele. Primers MK_wt_F and MK_wt_R produced an amplicon only for the wild type allele.

## Data Availability

Variants were submitted to dbSNP database (http://www.ncbi.nlm.nih.gov/SNP/) referred to as ss5015497294 and ss5015497295 (*Felis catus 9.0*; NC_018726.3:g. 174882895_174886198delins108). WGS data of the four Munchkin cats and eight controls were deposited in NCBI Sequence Read Archive (http://www.ncbi.nlm.nih.gov/sra) under the project number PRJNA505483 (SAMN10449164, SAMN10449165, SAMN10449169, SAMN10449170, SAMN14103114, SAMN14103116, SAMN14103119, SAMN14103120, SAMN14103121, SAMN14103123, SAMN14103124, SAMN14103127). Further WGS data for eight controls were retrieved from Sequence Read Archive (SRA, NCBI).

## Abbreviations

SNPs: Single nucleotide polymorphisms
FCA: Feline chromosome autosome
UGDH: UDP-Glucose 6-dehydrogenase
TICA: The International Cat Association
GWAS: Genome-wide association study
CT: Computed tomography
WGS: Whole genome sequencing
mm: Millimeter
In: Inch
Mb: Megabase
bp: Base pairs
UTR: Untranslated region
Indel: Insertion-deletion
aa: Amino acid
cDNA: Complementary DNA
ACAN: Aggrecan
HSPG: Heparan sulfate proteoglycan
UDP-GlcA: UDP-glucuronic acid
Fgf: Fibroblast growth factor
Hh: Hedgehog
TGFß: Transforming growth factor-ß
Wg,Wnt: Wingless
IACUC: The Institutional Animal Care and Use Committee
EDTA: Ethylenediaminetetraacetate
mAs: Milliampere seconds
kV: Kilovolt
DNA: Deoxyribonucleic acid
RNA: Ribonucleic acid
PCR: Polymerase chain reaction
mM: Millimolar
pmol: Picomol
KASP: Kompetitive Allele Specific PCR
ORF: Open reading frame finder
Min: Minute
TL: Total lengths
DL: Diaphyseal lengths
DD: Diaphyseal diameters
cm: Centimeters
wt: Wild type
sMc: Standard Munchkin cat
