# Supplementary material for: A structural UGDH variant associated with standard Munchkin cats
Source: BMC Genet. 2020 Jun 30;21:67. doi: 10.1186/s12863-020-00875-x (PMC7325026; doi:10.1186/s12863-020-00875-x)
Supplement: Supplementary file 2 — Additional file 2. Comparison of the length measurements for the long bones using CT images. A 4-year old male standard Munchkin cat is compared with an adult female domestic cat. Total (TL) and relative leg lengths, diaphyseal lengths (DL), and diaphyseal diameters (DD) in centimeters (cm), and inches (in) of front and hind limbs are shown. [file 12863_2020_875_MOESM2_ESM.docx]

**Additional file 2. Comparison of the length measurements for the long bones using CT images.** A 4-year old male standard Munchkin cat is compared with an adult female domestic cat. Total (TL) and relative leg lengths, diaphyseal lengths (DL), and diaphyseal diameters (DD) in centimeters (cm), and inches (in) of front and hind limbs are shown.

| Bone of the leg | Standard Munchkin cat | | | | Domestic cat | | | |
| --- | --- | --- | --- | --- | --- | --- | --- | --- |
|  | TL | TL in relation to leg length | DL | DD | TL | TL in relation to leg length | DL | DD |
| Front limbs | | | | | | | | |
| Humerus | 6.3 cm  (2.48 in) | 40.65 % | 4.9 cm  (1.90 in) | 0.8 cm  (0.31 in) | 8.9 cm  (3.50 in) | 40.09 % | 8.1 cm  (3.19 in) | 0.7 cm  (0.28 in) |
| Radius | 5.0 cm  (1.97 in) |  | 4.5 cm  (1.77 in) | 0.8 cm  (0.31 in) | 8.6 cm  (3.39 in) |  | 8.1 cm  (3.19 in) | 0.6 cm  (0.24 in) |
| Ulna | 6.5 cm  (2.56 in) | 41.94 % | 6.0 cm  (2.36 in) | 0,6 cm  (0,24 in) | 10.1 cm  (3.98 in) | 45.49 % | 9.2 cm  (3.62) | 0.7 cm  (0.28 in) |
| Metacarpalia I | 1.0 cm  (0.39 in) |  | 0.8 cm  (0.31 in) | 0.3 cm  (0.12 in) | 1.2 cm  (0.47 in) |  | 1.1 cm  (0.43 in) | 0.3 cm  (0.12 in) |
| Metacarpalia II | 2.7 cm  (1.06 in) |  | 2.2 cm  (0.87 in) | 0.4 cm  (0.16 in) | 2.9 cm  (1.14 in) |  | 2.6 cm  (1.02 in) | 0.3 cm  (0.12 in) |
| Metacarpalia III | 2.7 cm  (1.06 in) | 17.42 % | 2.2 cm  (0.87 in) | 0.4 cm  (0.16 in) | 3.2 cm  (1.26 in) | 14.41 % | 2.7 cm  (1.06 in) | 0.4 cm  (0.16 in) |
| Metacarpalia IV | 2.5 cm  (0.98 in) |  | 2.1 cm  (0.83 in) | 0.4 cm  (0.16 in) | 3.2 cm  (1.26 in) |  | 2.7 cm  (1.06 in) | 0.4 cm  (0.16 in) |
| Metacarpalia V | 2.4 cm  (0.94 in) |  | 2.1 cm  (0.83 in) | 0.4 cm  (0.16 in) | 2.6 cm  (1.02 in) |  | 2.3 cm  (0.91 in) | 0.3 cm  (0.12 in) |
| TL of humerus, ulna and metacarpalia III | 15.5 cm  (6.10 in) | | | | 22.2 cm  (8.74 in) | | | |
| Hind limbs | | | | | | | | |
| Femur | 7.5 cm  (2.95 in) | 38.46 % | 6.3 cm  (2.48 in) | 1.1 cm  (0.43 in) | 10.2 cm  (4.02 in) | 40.47 % | 9.3 cm  (3.66 in) | 0.9 cm  (0.35 in) |
| Tibia | 7.3 cm  (2.87 in) | 37.43% | 6.3 cm  (2.48 in) | 0.7 cm  (0.28 in) | 10.4 cm  (4.09 in) | 41.27 % | 9.8 cm  (3.86 in) | 0.8 cm  (0.31 in) |
| Metatarsalia II | 4.5 cm  (1.77 in) |  | 4.2 cm  (1.65 in ) | 0.4 cm  (0.16 in) | 4.5 cm  (1.77 in) |  | 4.2 cm  (1.65 in ) | 0.3 cm  (0.12 in) |
| Metatarsalia III | 4.7 cm  (1.85 in) | 24.10 % | 4.3 cm  (1.69 in) | 0.4 cm  (0.16 in) | 4.6 cm  (1.81 in) | 18.25 % | 4.3 cm  (1.69 in) | 0.4 cm  (0.16 in) |
| Metatarsalia IV | 4.7 cm  (1.85 in) |  | 4.3 cm  (1.69 in) | 0.5 cm  (0.20 in) | 4.6 cm  (1.81 in) |  | 4.3 cm  (1.69 in) | 0.4 cm  (0.16 in) |
| Metatarsalia V | 4.6 cm  (1.81 in) |  | 4.2 cm  (1.65 in ) | 0.4 cm  (0.16 in) | 4.5 cm  (1.77 in) |  | 4.2 cm  (1.65 in ) | 0.4 cm  (0.16 in) |
| TL of femur, tibia and metatarsalia III | 19.5 cm (7.68 in) | | | | 25.2 cm (9.92 in) | | | |
