# Supplementary material for: A structural UGDH variant associated with standard Munchkin cats
Source: BMC Genet. 2020 Jun 30;21:67. doi: 10.1186/s12863-020-00875-x (PMC7325026; doi:10.1186/s12863-020-00875-x)
Supplement: Supplementary file 4 — Additional file 4 Case-control study for the standard Munchkin cat phenotype using 14 SNPs on feline chromosome B1. The SNPs B1_5 to B1_14 are located within the significantly genome-wide associated region at 168 to 184 Mb. SNP IDs with their nomenclature for Felis catus 8.0 and (Felis catus 9.0), odds ratios with their lower and upper confidence intervals (CL), χ2-square values for the genotypic and allelic distribution as well as the corresponding –log10P-values are shown. The most significant values are printed in bold. [file 12863_2020_875_MOESM4_ESM.docx]

**Additional file 4. Case-control study for the standard Munchkin cat phenotype using 14 SNPs on feline chromosome B1.** The SNPs B1_5 to B1_14 are located within the significantly genome-wide associated region at 168 to 184 Mb. SNP IDs with their nomenclature for *Felis catus* 8.0 and (*Felis catus* 9.0), odds ratios with their lower and upper confidence intervals (CL), χ^2^-square values for the genotypic and allelic distribution as well as the corresponding –log_10_P-values are shown. The most significant values are printed in bold.

| SNP ID | Nomenclature for *Felis catus* 8.0 (*Felis catus* 9.0) | Odds ratio | Lower CL | Upper CL | χ^2^-square genotype | –log_10_P genotype | χ^2^-square allele | –log_10_P allele |
| --- | --- | --- | --- | --- | --- | --- | --- | --- |
| B1_1 | g.146611755C>T  (g.148289062C>T) | 2.50 | 0.6340 | 9.86 | 6.24 | 1.35 | 1.81 | 0.75 |
| B1_2 | g.150039652C>T  (g.151735648C>T) | 0.00 | . | . | 0.11 | 0.13 | 0.11 | 0.13 |
| B1_3 | g.151361269C>T  (g.153052513C>T) | 0.00 | . | . | 0.10 | 0.12 | 0.10 | 0.12 |
| B1_4 | g.156735182C>T  (g.158354497C>T) | 0.00 | . | . | 0.10 | 0.13 | 0.10 | 0.13 |
| B1_5 | g.168702941C>T  (g.170291080C>T) | 79.00 | 14.37 | 434.40 | 88.00 | 19.10 | 64.50 | 15.02 |
| B1_6 | g.170354472G>A  (g.171942256G>A) | 0.08 | 0.02 | 0.24 | 49.53 | 10.76 | 27.24 | 6.75 |
| B1_7 | g.171844393T>G  (g.173436205T>G) | 0.02 | 0.00 | 0.15 | 38.41 | 9.24 | 37.09 | 8.95 |
| B1_8 | g.172540503G>C  (g.174133775G>C) | 0.03 | 0.01 | 0.10 | 58.53 | 13.70 | 53.92 | 12.68 |
| **B1_9** | **g.173759872A>G**  **(g.175349169A>G)** | **151.00** | **16.78** | **1358.84** | **73.68** | **17.04** | **69.51** | **16.12** |
| **B1_10** | **g.174407393T>C**  **(g.175998162T>C)** | **0.01** | **0.00** | **0.06** | **74.59** | **17.24** | **69.87** | **16.20** |
| B1_11 | g.181992192T>C  (g.183566778T>C) | 0.04 | 0.00 | 0.36 | 17.14 | 4.46 | 16.70 | 4.36 |
| B1_12 | g.182422400C>T  (g.184002073C>T) | 14.60 | 2.26 | 94.39 | 13.30 | 3.58 | 12.89 | 3.48 |
| B1_13 | g.183810893A>G  (g.185398488A>G) | 14.00 | 2.16 | 90.54 | 12.69 | 3.44 | 12.29 | 3.34 |
| B1_14 | g.184508825C>T  (g.186099019C>T) | 12.40 | 1.92 | 80.27 | 11.08 | 3.06 | 10.69 | 2.97 |
