# Supplementary material for: A structural UGDH variant associated with standard Munchkin cats
Source: BMC Genet. 2020 Jun 30;21:67. doi: 10.1186/s12863-020-00875-x (PMC7325026; doi:10.1186/s12863-020-00875-x)
Supplement: Supplementary file 5 — Additional file 5 Haplotype analysis using SNPs B1_9 (g.173759872A > G) and B1_10 (g.174407393 T > C) as well as the indel (NC_018726.2:g.173294289_173297592delins108) on feline chromosome B1 (Felis catus 8.0). The haplotypes with their frequencies, χ2-statistics and P-values are shown. [file 12863_2020_875_MOESM5_ESM.docx]

**Additional file 5. Haplotype analysis using SNPs B1_9 (g.173759872A>G) and B1_10 (g.174407393T>C) as well as the indel (NC_018726.2:g.173294289_173297592delins108) on feline chromosome B1 (*Felis catus* 8.0).** The haplotypes with their frequencies, χ^2^- statistics and P-values are shown.

| Haplotype frequencies | Haplotype  indel - B1_9- B1_10 | Wildtype/  non-standard Munchkin cat | Standard Munchkin cats | χ^2^-value | P-value |
| --- | --- | --- | --- | --- | --- |
| 0.944 | wt-A-T | 1.00000 | 0.50 | 239.8799 | <0.0001 |
| 0.056 | Indel-G-C | 0.00000 | 0.50 | 11.1830 | 0.0008 |
