# Supplementary figures and images for: A structural UGDH variant associated with standard Munchkin cats
Source: BMC Genet. 2020 Jun 30;21:67. doi: 10.1186/s12863-020-00875-x (PMC7325026; doi:10.1186/s12863-020-00875-x)

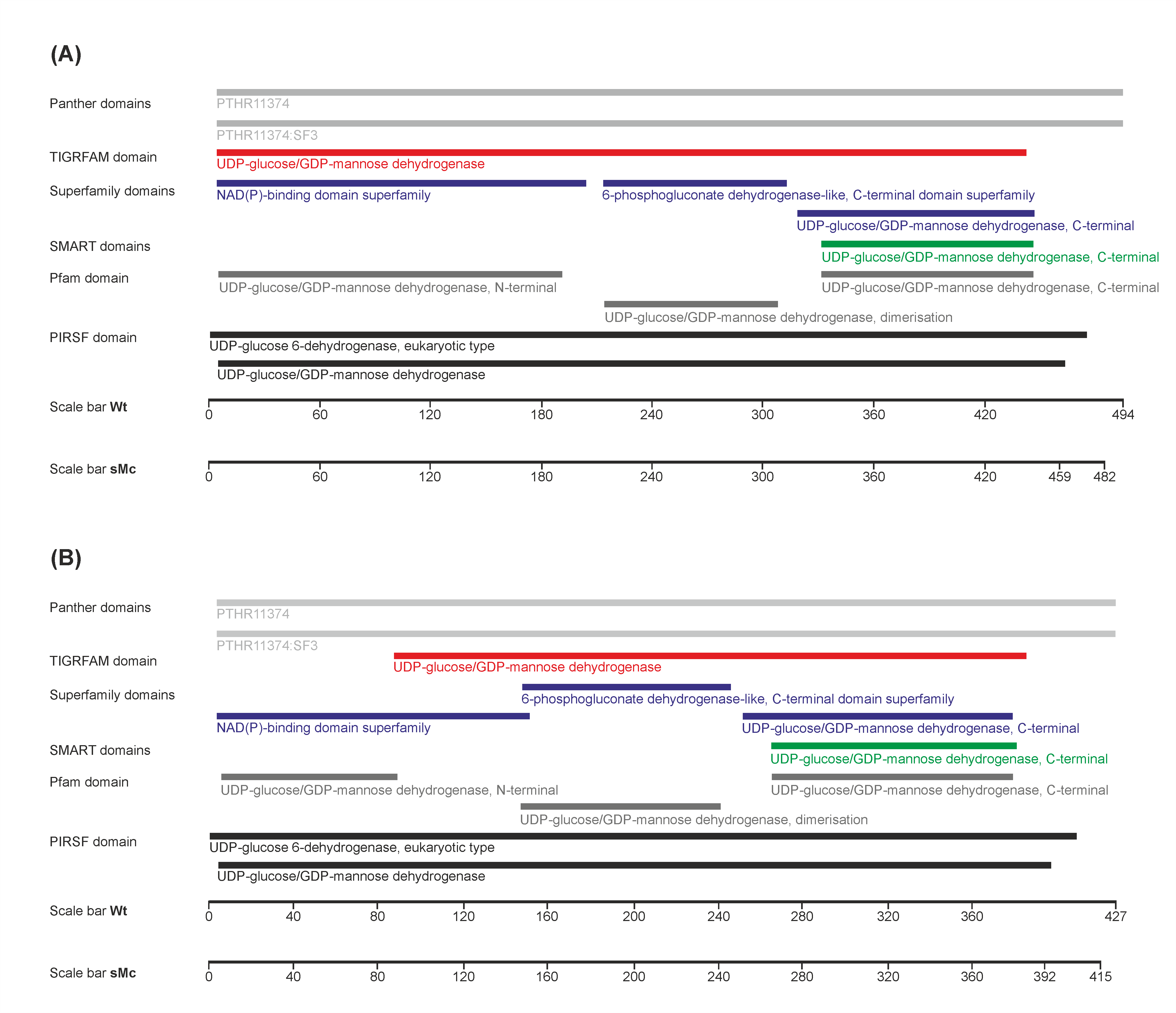

Supplement: Supplementary file 10 — Additional file 10 Domains predicted by InterProScan. (A) Predicted protein domains of transcript UGDH-201 (ENSFCAT00000009602.6) and (B) UGDH-202 (ENSFCAT000000557945.1) based on Felis catus 9.0 (Ensembl protein summary) are shown. Wild type cat (wt) protein length are compared to standard Munchkin cat (sMc) length. [file 12863_2020_875_MOESM10_ESM.tif]
