# Supplementary material for: A structural UGDH variant associated with standard Munchkin cats
Source: BMC Genet. 2020 Jun 30;21:67. doi: 10.1186/s12863-020-00875-x (PMC7325026; doi:10.1186/s12863-020-00875-x)
Supplement: Supplementary file 12 — Additional file 12. Number of animals and breeds included in this study. Standard Munchkin cat subtypes, non-standard Munchkin cat subtypes and controls from various breeds are displayed. [file 12863_2020_875_MOESM12_ESM.docx]

**Additional file 12. Number of animals and breeds included in this study.** Standard Munchkin cat subtypes, non-standard Munchkin cat subtypes and controls from various breeds are displayed.

| **Animals** | **Number of animals** |
| --- | --- |
|  |  |
| **Standard Munchkin cat subtypes** |  |
| Genetta | 10 |
| Napoleon | 2 |
| Dwelf | 1 |
| Bambino | 10 |
| Lambkin | 1 |
| longhaired Munchkin cat | 11 |
| shorthaired Munchkin cat | 7 |
| **Non-standard Munchkin cat subtypes** |  |
| Bambino | 6 |
| Genetta | 2 |
| longhaired Munchkin cat | 1 |
| shorthaired Munchkin cat | 6 |
| **Controls** |  |
| British Shorthair | 18 |
| Bengal | 19 |
| Ragdoll | 2 |
| Exotic Shorthair | 2 |
| Don Sphinx | 5 |
| Birman | 2 |
| Chartreux | 3 |
| Maine Coon | 13 |
| Norwegian Forest | 10 |
| Persian | 23 |
| Russian Blue | 1 |
| Scottish Fold | 8 |
| Siamese | 17 |
| Siberian | 2 |
| Turkish Angora | 2 |
| Oriental Shorthair | 48 |
| Domestic shorthair | 28 |
| Total | 260 |
