# Supplementary material for: A structural UGDH variant associated with standard Munchkin cats
Source: BMC Genet. 2020 Jun 30;21:67. doi: 10.1186/s12863-020-00875-x (PMC7325026; doi:10.1186/s12863-020-00875-x)
Supplement: Supplementary file 13 — Additional file 13 Primer pairs used for haplotype analysis. The single nucleotide variants located on chromosome B1 were genotyped using a Kompetitive Allele Specific PCR (KASP) assay. The SNP ID, nomenclature of Felis catus 8.0 and 9.0, accession numbers, sequences of forward and reverse primers, annealing temperature (AT), as well as the number of cycles are shown. [file 12863_2020_875_MOESM13_ESM.docx]

**Additional file 13. Primer pairs used for haplotype analysis.** The single nucleotide variants located on chromosome B1 were genotyped using a Kompetitive Allele Specific PCR (KASP) assay. The SNP ID, nomenclature of *Felis catus 8.0* and *9.0*, accession numbers, sequences of forward and reverse primers, annealing temperature (AT), as well as the number of cycles are shown.

| SNP ID | Nomenclature for  *Felis catus* 8.0  (*Felis catus* 9.0) | Accession number | Forward primers (5’-3’) | Reverse primer (5’-3’) | AT (°C) | Number  of cycles |
| --- | --- | --- | --- | --- | --- | --- |
| B1_1 | g.146611755C>T  (g.148289062C>T) | ss5015497298 | GCAACGGAGATGCAGGTCCG-FAM  GGCAACGGAGATGCAGGTCCA-VIC | CACCAGGTGAGCGAAATCTGCAATA | 61 | 29 |
| B1_2 | g.150039652C>T  (g.151735648C>T) | ss5015497292 | GTGAAGGTACCAGGACAGTTAC-FAM  GTGAAGGTACCAGGACAGTTAT-VIC | GATCAACTCCASCTTCGTGGGCAT | 61 | 29 |
| B1_3 | g.151361269C>T  (g.153052513C>T) | ss5015497301 | ACCCAGGTGCCCCTAGAATTTC-FAM  CACCCAGGTGCCCCTAGAATTTT-VIC | CGACTTGCACTCTCTCACTCTCAAA | 61 | 29 |
| B1_4 | g.156735182C>T  (g.158354497C>T) | ss5015497296 | GACCTCCTTGGTTGATGTTTTGC-FAM  GGACCTCCTTGGTTGATGTTTTGT-VIC | CAGGAAGCACAAAGAGACTTTAAGGAAA | 61 | 29 |
| B1_5 | g.168702941C>T  (g.170291080C>T) | ss5015497291 | GAAAGCTTTAAGTAAAAGTTTTATTAAAGACG- FAM  GGAAAGCTTTAAGTAAAAGTTTTATTAAAGACA-VIC | CTGTAAATTCCAATATGCCAGTCTGACTT | 61 | 29 |
| B1_6 | g.170354472G>A  (g.171942256G>A) | ss5015497290 | GGTATAGATGACAGGATTGCTAGTG-FAM  AAGGTATAGATGACAGGATTGCTAGTA-VIC | GAACTTGGGTAATGGGAAGCATATTCAAT | 61 | 29 |
| B1_7 | g.171844393T>G  (g.173436205T>G) | ss5015497303 | GAGAACTAGGCTGTCCAGGACA-FAM  AGAACTAGGCTGTCCAGGACC-VIC | CCCATGCTGAATTTTTAGACAGTTGTGTT | 61 | 29 |
| B1_8 | g.172540503G>C  (g.174133775G>C) | ss5015497305 | TCTAGCCTCCAGAACTGTGAG-FAM  CTTCTAGCCTCCAGAACTGTGAC-VIC | AACTGGGTGGCTTAAACAGCAGAAATATA | 61 | 41 |
| B1_9 | g.173759872A>G  (g.175349169A>G) | ss5015497300 | ATTTTATTTTTTGCGACTTATAAACTTCCTTAT-FAM  TTATTTTTTGCGACTTATAAACTTCCTTAC-VIC | GCATATCTGCATAGAAAGTCCAAAAGATTT | 61 | 29 |
| B1_10 | g.174407393T>C  (g.175998162T>C) | ss5015497299 | ACTGTACAGCCGTAGGCAGGA-FAM  CTGTACAGCCGTAGGCAGGG-VIC | GACATTCATCCTGTCTGCCCTGATA | 61 | 29 |
| B1_11 | g.181992192T>C  (g.183566778T>C) | ss5015497302 | TCCCAGCTTTGCCACCCA-FAM  CTTCCCAGCTTTGCCACCCG-VIC | AAGTAAAGTAGAAAAGGACACACCTCCAA | 61 | 29 |
| B1_12 | g.182422400C>T  (g.184002073C>T) | ss5015497297 | CCAATTGGTTTATTCCCATTACTCCC-FAM  CCAATTGGTTTATTCCCATTACTCCT-VIC | GCTACCCTGATTGGCACTCAATGTA | 61 | 29 |
| B1_13 | g.183810893A>G  (g.185398488A>G) | ss5015497293 | AGATACAACTAAGTTAAATTTCAGTATGGTTTA-FAM  GATACAACTAAGTTAAATTTCAGTATGGTTTG-VIC | CATACTGCTTTTGGCTCATAGATTTGGTT | 61 | 32 |
| B1_14 | g.184508825C>T  (g.186099019C>T) | ss5015497304 | AATAGTTSCTGTTACTGGAAGGTCG-FAM  AAAATAGTTSCTGTTACTGGAAGGTCA-VIC | GCTTCGATTGACTGTCAAATAATGTTTCAA | 61 | 29 |
