# Supplementary material for: A structural UGDH variant associated with standard Munchkin cats
Source: BMC Genet. 2020 Jun 30;21:67. doi: 10.1186/s12863-020-00875-x (PMC7325026; doi:10.1186/s12863-020-00875-x)
Supplement: Supplementary file 14 — Additional file 14 Primer pairs used for complementary DNA amplification of UGDH. PCR was done to test if the 108 bp insert is transcribed or not transcribed based on Felis catus 9.0. PCR-type 1 produces an expected amplicon of 510 bp for the mutant allele if the insertion is transcribed, and a 402 bp for the mutant allele if the insertion is not transcribed, as well as 2514 bp for the wild type allele. PCR-type 2, spanning from exon 10 (UGDH-201 (ENSFCAT00000009602.6)) or exon 9 (UGDH-202 (ENSFCAT00000055794.2)) to the 108 bp insert, should have an expected amplicon size of 110 bp for the mutant allele if the insertion is transcribed. PCR-type 3 produces an amplicon with 185 bp in standard Munchkin cats, and controls for the wild type allele. [file 12863_2020_875_MOESM14_ESM.docx]

**Additional file 14. Primer pairs used for complementary DNA amplification of *UGDH*.** PCR was done to test if the 108 bp insert is transcribed or not transcribed based on *Felis catus 9.0*. PCR-type 1 produces an expected amplicon of 510 bp for the mutant allele if the insertion is transcribed, and a 402 bp for the mutant allele if the insertion is not transcribed, as well as 2,514 bp for the wild type allele. PCR-type 2, spanning from exon 10 (UGDH-201 (ENSFCAT00000009602.6)) or exon 9 (UGDH-202 (ENSFCAT00000055794.2)) to the 108 bp insert, should have an expected amplicon size of 110 bp for the mutant allele if the insertion is transcribed. PCR-type 3 produces an amplicon with 185 bp in standard Munchkin cats, and controls for the wild type allele.

| PCR-type | Primer pair (Revers: R, Forward: F) | Located in *UGDH-201* | Located in *UGDH-202* | Primer sequence (5’-3’) | AT (°C) | Expected products | | | | | |
| --- | --- | --- | --- | --- | --- | --- | --- | --- | --- | --- | --- |
|  |  |  |  |  |  | Wild type cat | | Standard Munchkin cat | | | |
|  |  |  |  |  |  |  | | with 108 bp insertion | | | without 108 bp insertion |
| 1 | FCA_B1_cUGDH_F1 | exon 10 | exon 9 | GCCAGCCTTTATCTTTGATGG | 54 | 2,514 bp | | 510 bp | | 402 bp | |
|  | FCA_B1_cUGDH_R1 | 3’ UTR | 3’ UTR | TCTGGTGTTTGTCTCTTTCTGG |  |  | |  |  | | |
| 2 | FCA_B1_cUGDH_F2 | exon 10 | exon 9 | TGAAGCCAGCCTTTATCTTTG | 54 | - | | 110 bp | | - | |
|  | FCA_B1_cUGDH_R2 | 108 bp insertion | 108 bp insertion | TCTCTCTATAATCCGGGAAGC |  |  | |  |  | | |
| 3 | FCA_B1_cUGDH_F3 | exon 10 | exon 9 | AGCCAGCCTTTATCTTTGATG | 55 | 185 bp | | 185 bp | | 185 bp | |
|  | FCA_B1_cUGDH_R3 | exon 11 | exon 10 | CTATACTCTGGGTTTCTTGTTCG |  |  |  | |  | | |
