# Supplementary material for: A structural UGDH variant associated with standard Munchkin cats
Source: BMC Genet. 2020 Jun 30;21:67. doi: 10.1186/s12863-020-00875-x (PMC7325026; doi:10.1186/s12863-020-00875-x)
Supplement: Supplementary file 15 — Additional file 15. Primer pairs used for complementary DNA amplification spanning exon 10 (ENSFCAT00000009602.6) or exon 9 (ENSFCAT00000055794.2) to 3’UTR. All PCR-products were present in the wild type allele. PCR-type, primer pairs, sequences of forward and reverse primer, annealing temperatures (AT), and expected product sizes (bp) are shown. [file 12863_2020_875_MOESM15_ESM.docx]

**Additional file 15. Primer pairs used for complementary DNA amplification spanning exon 10 (ENSFCAT00000009602.6) or exon 9 (ENSFCAT00000055794.2) to 3´UTR.** All PCR-products were present in the wild type allele. PCR-type, primer pairs, sequences of forward and reverse primer, annealing temperatures (AT), and expected product sizes (bp) are shown.

| PCR-type | Primer pair  (Reverse: R; Forward: F) | Primer sequence (5’-3’) | AT (°C) | Expected product size (bp) |
| --- | --- | --- | --- | --- |
| 4 | FCA_B1_Wt_UGDH_F1 | ATCCACCGAACAAGAAACCC | 55 | 582 |
|  | FCA_B1_Wt_UTR_cUGDH_R1 | CAAGGCCCCATTCTATCCAC |  |  |
| 5 | FCA_B1_Wt_UTR_cUGDH_F2 | TAAACCGTGAAATTGCTGTCC | 56 | 431 |
|  | FCA_B1_Wt_UTR_cUGDH_R2 | AGCAGTGATGACATTCTTGGG |  |  |
| 6 | FCA_B1_Wt_UTR_cUGDH_F3 | GCTTCCATGTTACAAGTGACC | 55 | 711 |
|  | FCA_B1_Wt_UTR_cUGDH_R3 | TCAGACTGTCGTGACTCATTTG |  |  |
| 7 | FCA_B1_Wt_UTR_cDNA_F4 | TTGCGTGAGTGTATTTTCTGTC | 55 | 421 |
|  | FCA_B1_Wt_UTR_cDNA_R4 | CCATGAGCTTTGATTTGCAGG |  |  |
| 8 | FCA_B1_Wt_UTR_cDNA_F5 | CTGGTGCCTGCAAATCAAAG | 55 | 576 |
|  | FCA_B1_cUGDH_R1 | TCTGGTGTTTGTCTCTTTCTGG |  |  |
