# Supplementary material for: A structural UGDH variant associated with standard Munchkin cats
Source: BMC Genet. 2020 Jun 30;21:67. doi: 10.1186/s12863-020-00875-x (PMC7325026; doi:10.1186/s12863-020-00875-x)
Supplement: Supplementary file 16 — Additional file 16. Primer pairs used for validation and Sanger sequencing. Validation of the indel was done using duplex PCR (MK_wt_F, MK_wt_R and MK_del_R), amplicons for Sanger sequencing were generated with primer pairs MK_wt_F, and MK_del_R. [file 12863_2020_875_MOESM16_ESM.docx]

**Additional file 16. Primer pairs used for validation and Sanger sequencing.** Validation of the indel was done using duplex PCR (MK_wt_F, MK_wt_R and MK_del_R), amplicons for Sanger sequencing were generated with primer pairs MK_wt_F, and MK_del_R.

| Primer pair  (Reverse: R; Forward: F) | Polymorphism | Primer sequence (5’-3’) | AT (°C) | Number  of cycles |
| --- | --- | --- | --- | --- |
| MK_del_R | g.173294289_173297592del | AGCTCATTTTCACTTCAGGATCT | 59 | 40 |
| MK_wt_F | g.173294289_173297592del | ACTTCCAAGGCGTAAACACATTT | 59 | 40 |
| MK_wt_R | g.173294289_173297592del | GCATAGATTTCAGGAGTTCAGGC | 59 | 40 |
